# Opportunities and Challenges in Functional Genomics Research in Osteoporosis: Report From a Workshop Held by the Causes Working Group of the Osteoporosis and Bone Research Academy of the Royal Osteoporosis Society on October 5th 2020

**DOI:** 10.3389/fendo.2020.630875

**Published:** 2021-02-15

**Authors:** Jonathan H. Tobias, Emma L. Duncan, Erika Kague, Chrissy L. Hammond, Celia L. Gregson, Duncan Bassett, Graham R. Williams, Josine L. Min, Tom R. Gaunt, David Karasik, Claes Ohlsson, Fernando Rivadeneira, James R. Edwards, Fadil M. Hannan, John P. Kemp, Sophie J. Gilbert, Nerea Alonso, Neelam Hassan, Juliet E. Compston, Stuart H. Ralston

**Affiliations:** ^1^ Musculoskeletal Research Unit, Translational Health Sciences, Bristol Medical School, University of Bristol, Bristol, United Kingdom; ^2^ MRC Integrative Epidemiology Unit, Bristol Medical School, University of Bristol, Bristol, United Kingdom; ^3^ Department of Twin Research & Genetic Epidemiology, School of Life Course Sciences, King’s College London, London, United Kingdom; ^4^ School of Physiology, Pharmacology and Neuroscience, University of Bristol, Bristol, United Kingdom; ^5^ Molecular Endocrinology Laboratory, Department of Metabolism, Digestion & Reproduction, Hammersmith Hospital, Imperial College London, London, United Kingdom; ^6^ Azrieli Faculty of Medicine, Bar-Ilan University, Safed, Israel; ^7^ Center for Bone and Arthritis Research, Sahlgrenska Academy, University of Gothenburg, Gothenburg, Sweden; ^8^ Department of Internal Medicine, Erasmus University Medical Center, Rotterdam, Netherlands; ^9^ Botnar Research Centre, Nuffield Department of Orthopaedics, Rheumatology and Musculoskeletal Sciences, University of Oxford, Oxford, United Kingdom; ^10^ Nuffield Department of Women’s & Reproductive Health, University of Oxford, Oxford, United Kingdom; ^11^ University of Queensland Diamantina Institute, University of Queensland, Woolloongabba, Queensland, QLD, Australia; ^12^ Biomechanics and Bioengineering Centre Versus Arthritis, Cardiff School of Biosciences, Cardiff, United Kingdom; ^13^ Centre for Genomic and Experimental Medicine, Institute of Genetics and Molecular Medicine, University of Edinburgh, Edinburgh, United Kingdom; ^14^ Department of Medicine, Cambridge Biomedical Campus, University of Cambridge, Cambridge, United Kingdom

**Keywords:** genome-wide association study, bone mineral density, mouse model, zebrafish, “omics” data

## Abstract

The discovery that sclerostin is the defective protein underlying the rare heritable bone mass disorder, sclerosteosis, ultimately led to development of anti-sclerostin antibodies as a new treatment for osteoporosis. In the era of large scale GWAS, many additional genetic signals associated with bone mass and related traits have since been reported. However, how best to interrogate these signals in order to identify the underlying gene responsible for these genetic associations, a prerequisite for identifying drug targets for further treatments, remains a challenge. The resources available for supporting functional genomics research continues to expand, exemplified by “multi-omics” database resources, with improved availability of datasets derived from bone tissues. These databases provide information about potential molecular mediators such as mRNA expression, protein expression, and DNA methylation levels, which can be interrogated to map genetic signals to specific genes based on identification of causal pathways between the genetic signal and the phenotype being studied. Functional evaluation of potential causative genes has been facilitated by characterization of the “osteocyte signature”, by broad phenotyping of knockout mice with deletions of over 7,000 genes, in which more detailed skeletal phenotyping is currently being undertaken, and by development of zebrafish as a highly efficient additional *in vivo* model for functional studies of the skeleton. Looking to the future, this expanding repertoire of tools offers the hope of accurately defining the major genetic signals which contribute to osteoporosis. This may in turn lead to the identification of additional therapeutic targets, and ultimately new treatments for osteoporosis.

## Introduction

This perspective article provides a viewpoint on the opportunities and challenges in functional genomics research in osteoporosis, synthesizing the content of a recent workshop of invited experts. This was held to provide a blueprint for research and funding proposals in this area, with the ultimate aim of translating discoveries from human genetic studies into new therapies for patients with osteoporosis.

### The Need for New Osteoporosis Therapies

Anti-resorptive drugs are the mainstay of treatment in osteoporosis. Despite being widely used, adherence rates in the US (1) and UK (2) are decreasing, and these agents have several limitations including poor tolerability in the case of oral bisphosphonates, and risk of rare adverse effects including osteonecrosis of the jaw and atypical femoral fractures. Anabolic therapies for osteoporosis may offer certain advantages, including greater efficacy than some anti-resorptives, and lack of the adverse effects associated with suppression of bone resorption. However, currently available anabolic drugs are costly, and need to be given by injection, limiting their use to a small fraction of patients with osteoporosis. Thus, there is an urgent need for a low cost, ideally orally active, anabolic therapy for osteoporosis.

### The Potential of Human Genetic Studies for Drug Discovery in Osteoporosis

#### Rare Bone Diseases

The heritable condition of increased bone fragility, Osteogenesis Imperfecta (OI), was the first bone disorder to have the underlying genetic mutation identified. Linkage analysis identified the *COLIA1* and *COLIA2* genes as candidate loci for the disease and soon after this, various mutations were identified in both genes as a common cause of OI (3, 4). Many other mutations underlying OI have since been identified. Though most of these affect genes which are involved with post-translational modifications of type 1 collagen, some affect osteoblast differentiation and function (5). However, findings from OI genetics studies are yet to provide tangible opportunities for developing new osteoporosis treatments.

In contrast, studies of rare bone diseases associated with low or high bone mass (HBM) have provided the basis for a new osteoporosis therapy in the form of Romosozumab, following the discovery that sclerostin – LRP5 regulation of the Wnt signaling pathway plays a major role in bone biology. Romosozumab is an anti-sclerostin antibody, which has recently been developed as anabolic treatment for osteoporosis, and is now widely available. Sclerostin, encoded by *SOST*, was initially identified from the study of patients with the heritable HBM disorder, sclerosteosis (6). Several other genes underlying HBM have also been identified, representing possible therapeutic targets for additional anabolic therapies. These include a recently identified inactivating mutation in *SMAD9*, which encodes an inhibitor of BMP signaling (7). Further analysis of existing case collections of familial HBM may offer the opportunity to identify further possible drug targets.

Advances in the understanding of other rare bone diseases with a genetic component, such as pregnancy associated osteoporosis (8), might yield targets for new drug design or re-purposing existing drugs, which if successful might also apply to treating osteoporosis (9). In considering the pipeline for similar discoveries, the classification of genetic skeletal disorders lists 437 genes for 425 different diseases (10). For example, achondroplasia caused by a mutation in the fibroblast growth factor receptor 3 gene, a negative regulator of bone growth, now has an natriuretic peptide receptor 2 (NPR2) agonist (Vosoritide) developed as treatment (11). Of interest, factors from the C-type natriuretic peptide signaling pathway have previously been associated with human stature (12), among which a locus within the gene encoding NPR3, with which NPR2 complexes, was also recently identified in a genome wide association study (GWAS) of extreme high bone mass (13). Rare disorders associated with impaired osteoclastic bone resorption may also have utility in treating osteoporosis, exemplified by pycnodysostosis caused by cathepsin K deficiency (14), for which the inhibitor Odanocatib was developed as a new anti-resorptive treatment for osteoporosis. In addition, drug-repurposing may provide novel means of treating rare bone disorders. For example, palovarotene, a retinoic acid receptor gamma (RAR-γ) agonist developed for use in emphysema, was found to be efficacious in an animal model of fibrodysplasia ossificans progressiva (FOP), and is now in phase 3 clinical trials (15). In addition, Fresolimumab, a human monoclonal antibody directed against transforming growth factor B2, developed for treating idiopathic pulmonary fibrosis, is currently being examined to treat OI (ClinicalTrials.gov identifier NCT03064074).

#### Genome Wide Association Studies (GWAS)

Many GWAS have been performed for endpoints related to osteoporosis-related phenotypes, including fractures (16), bone mineral density (BMD) as measured by DXA (17–19), as well as estimated by calcaneal ultrasound (eBMD) (20–22). These were undertaken by the GEnetic Factors for OSteoporosis Consortium (GEFOS), representing over 30 countries (http://www.gefos.org/). Whereas BMD represents an overall measure of bone quantity, GWAS have also been performed of endophenotypes related to cortical and trabecular bone as measured by peripheral quantitative computed tomography (pQCT) (23–26), and more recently high resolution (HR)-pQCT (27). Fracture risk can also reflect other characteristics such as bone shape and geometry, which have similarly been examined by GWAS (28, 29). To date, in contrast to the study of rare monogenic disorders, no GWAS of common variation in bone phenotypes has led to a new treatment for osteoporosis. That said, well powered GWAS have only been available in the relatively recent past, and the above GWAS have found genome-wide significant variants in genes coding for existing osteoporosis drug targets, e.g., romosozumab (*SOST*), denosumab (*RANKL*) and raloxifene (*ESR1*).

GWAS findings can also be helpful in predicting side-effects arising from the drug target in question having actions outside the skeleton. For example, a BMD GWAS signal related to *SOST* was used to examine potential cardiovascular toxicity of romosozumab (30). Several open-source data and analytical platforms, using published and unpublished GWAS summary datasets, have been developed to interrogate genetic correlations/causal effects in relation to thousands of traits and diseases, thereby predicting co-morbidities and extra-skeletal effects using a hypothesis-free approach. Examples include MR-Base (31), the polygenic risk score atlas (32), and LD-hub (33). Multiple risk factors for osteoporosis have also been scrutinized for causal associations using a Mendelian randomization (MR) framework (34, 35). For example, the GEFOS GWAS for fracture risk leveraged the MR approach to demonstrate, that among the recognized clinical risk factors, BMD is a “causally-related” determinant of fracture risk implying that targeting to increase BMD or prevent its loss is likely to be successful in decreasing fracture risk (16). This MR study also found that genetically-determined vitamin D levels are not causally related to fracture risk, supporting conclusions from clinical trials that vitamin D supplementation in “sufficient” individuals is ineffective in preventing fractures. Likewise, calcium intake was found to have no causal effect on fracture risk, suggesting an adverse risk/benefit ratio when the associated increased risk of coronary artery disease is taken into account (36, 37). GWAS findings may also have potential application as clinical risk prediction tools, as exemplified by a recent study examining implementation of a polygenic fracture risk score in combination with FRAX (38).

GWAS have been helpful in predicting the effectiveness of new drug therapies (39, 40). GWAS have also identified potential new drug targets in other musculoskeletal conditions. For example, GWAS in ankylosing spondylitis (AS) and psoriatic arthritis (PsA) identified IL23R, IL12B, and IL17A as associated loci, facilitating the development of ustekinumab, an IL12/23 inhibitor used in PsA, and secukinumab, an IL17A inhibitor used in both AS and PsA (41, 42).

Large well-powered GWAS often yield a multitude of genetic signals, but a major challenge is to map the association signals to the causal gene due to the correlated structure of the genome and to follow up those genetic signals from the point of view of functional studies. For example, the most recent eBMD GWAS from the UK Biobank Study, based on the whole cohort of around 425,000 individuals, identified 1103 independent association signals mapping to 515 loci (22). Combined with an earlier eBMD GWAS performed on a subset of the UK Biobank Study (20), these two studies investigated, and functionally annotated over 160 mouse lines with deletions of orthologous genes corresponding to associated GWAS loci, demonstrating the power of integrating disparate datasets from human GWAS and animal studies. Nevertheless, little functional information was obtained in the case of many of the genetic signals identified, due to lack of an available mouse model. For example, a signal associated with the *SMAD9* locus, described above, was initially discovered, but not interrogated further, in the original eBMD GWAS on 150,000 UK participants (20). The latter GWAS also identified a further signal, *B4GALNT3*, which was only later found to influence BMD by altering sclerostin levels, following a separate GWAS of serum sclerostin (43).

## Functional Genomics: *In Silico* Studies

As stated above, a major challenge in analyzing outputs of genetics studies is to identify the gene underlying the genetic association observed. Several platforms are available to interrogate outputs from GWAS studies, aiming to identify causal SNPs and the genes they affect. Different methods are often applied in parallel to identify potential functional effects of SNPs, map these to genes and investigate the function of candidate genes identified in this way. A range of sources of omic information used for interrogating genetic signals in relation to skeletal disorders have recently been integrated within the IFMRS knowledge portal [https://msk.hugeamp.org/ (44)].

### SNP-Based Analyses

Independent signals and lead variants are initially fine mapped across an identified locus using methods such as FINEMAP (45). Subsequently, identified SNPs are annotated according to their likelihood of exerting a functional effect. In the case of monogenic disorders, the underlying genetic variant is expected to alter protein function, for example as a consequence of a non-synonymous exon variant. In contrast, in GWAS, the variant is likely to affect gene expression, for example due to a base change affecting DNA binding of a transcriptional activating factor within the promoter region. Several different approaches for SNP annotation have been developed. For example, ENCODE, the encyclopedia of DNA elements (https://www.encodeproject.org/) provides a range of features which can be used to evaluate potential functional SNPs. Machine learning approaches have been used to predict functional effects using the most discerning features from ENCODE and other databases. These algorithms are trained on disease-causing mutations and assumed neutral variants, enabling the algorithms to classify SNPs as potentially deleterious or neutral. In non-coding regions, where most GWAS SNPs are located, sequence conservation has been found to be by far the most informative feature (46). However, such algorithms are not disease-specific, so whether this also applies to bone related conditions remains to be established. Besides ENCODE, several other strategies for SNP annotation have been developed. These include ATAC-seq to study intersections between SNPs and sites of open chromatin as previously identified in osteoblast cell lines (22) and mouse bone tissue (43), and Hi-C to interrogate 3D DNA interactions as previously characterized in osteoblasts (22).

### Gene-Based Analyses

Having identified lead SNP(s), the function of closest protein coding genes is explored to guide further follow up. Since bone-specific pathways are thought most likely to underlie skeletal phenotypes, if a gene is found to be expressed in bone, this is assumed to increase the likelihood that it underlies a given osteoporosis genetic association signal. This approach has been facilitated by description of the “osteocyte signature” (47), referring to the set of genes expressed preferentially in osteocytes, which was used to interrogate the genetic signals identified by Morris et al. (22). As described below, information from skeletal phenotyping of mouse lines can also help to identify genes which are likely to underlie genetic association signals relevant to osteoporosis, as are previous reports that the gene in question is related to a skeletal disorder in humans. In the case of genes not previously known to play a role in bone, methods such as DEPICT can be used to predict function based on relationships with known pathways (48).

### “Omics” Approaches to Map GWAS Signals to Specific Genes

One approach to mapping genetic signals to specific genes is to examine causal pathways between the genetic signal and the phenotype being studied, involving potential molecular mediators such as mRNA expression, protein expression, and DNA methylation levels. These molecular quantitative trait loci (QTLs) are generally classified into *cis*-acting, where the SNP is located nearby a gene or site or *trans*-acting, where the site or gene is located more distantly or on another chromosome. *Cis*-acting molecular QTLs tend to have larger effect sizes whereas *trans* effects have smaller effect sizes and require larger sample sizes to detect these associations. Large scale initiatives such as GTEX, eQTLGen (49), Genetics of DNA Methylation Consortium (GoDMC) (50) and SCALLOP (51) have been established to identify these small effects that might play a role in disease etiology.

**Figure 1:**
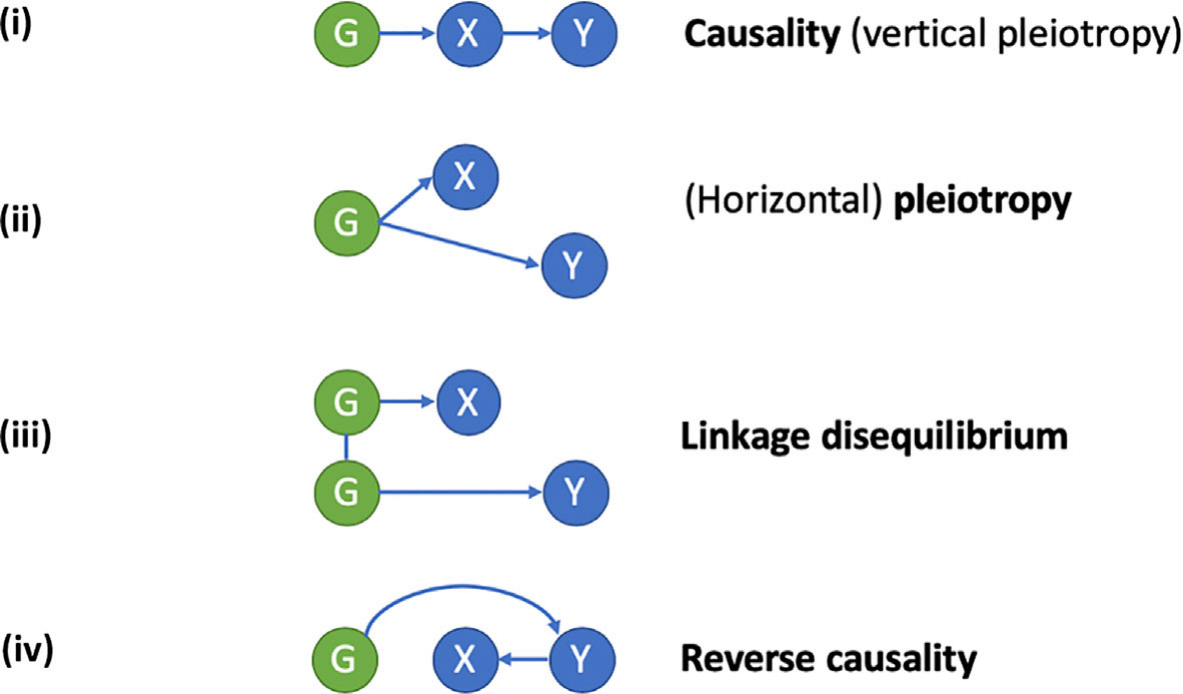
Applying a Mendelian randomization (MR) framework to study causal inferences in “omics” data. In conventional MR, a genetic instrument (G) is used as a proxy for an exposure (X), to study its relationship with a disease outcome (Y). A causal relationship of X on Y exists, if G is related to Y *via* its effects on X. An example is the use of genetic polymorphisms related to bone mineral density (BMD) to study the causal relationship between low BMD (X) and fracture risk (Y). When applied to “omics” data, X represents an intermediate molecular trait (i.e., mRNA, DNA methylation, or protein level) mediating the relationship between genotype (G) and disease outcome (Y). Since the intermediate trait is gene-specific, finding of a causal relationship is helpful in defining which gene (or regulatory element in the case of DNA methylation) underlies the association between G and Y. Causal inference using MR relies on the exclusion of horizontal pleiotropy, confounding by linkage disequilibrium and reverse causality. (i) Causality/vertical pleiotropy: G has a causal effect on intermediate molecular trait X, which in turn has a causal effect on Y. (ii) Horizontal pleiotropy: G has a causal effect on both X and Y *via* independent pathways. (iii) Linkage disequilibrium: G has a causal effect on X, but its relationship with Y is a consequence of linkage disequilibrium with a separate genetic variant causal for Y. (iv) Reverse causality: G has a causal effect on Y which subsequently alters X.

Co-localization studies across a range of disorders and phenotypes have been conducted to examine whether molecular QTLs share genetic variation with GWA signals, thereby linking a given genetic signal with the function of a specific gene. Evidence for a number of shared genetic factors between BMD GWA loci and protein quantitative trait loci (pQTLs) have been found in blood (52). However, whereas approaches such as co-localization analyses can be used to examine shared relationships between a given genetic locus, phenotype, and intermediary signal, these may not necessarily represent a causal pathway from the genetic signal to the phenotype. Approaches such as MR can be used to estimate causal effects (**Figure 1**), but require bi-directional analyses to exclude reverse causation, and in many cases will not exclude horizontal pleiotropy as an alternative explanation of co-localization (i.e., the genetic signal influences the phenotype being studied *via* an independent pathway to the gene showing related changes in expression). To improve reliability of MR, multiple independent genetic variants influencing a molecular trait can be employed, which should exhibit consistent causal effects (53). In a recent analysis combining MR and co-localization analysis to examine GWA with the plasma proteome, only a minority of associations were found to be causal, and mainly restricted to *cis*-associations (52). Similarly, the GoDMC study estimated the causal relationship between DNA methylation in blood and 116 complex traits. This study used multiple *cis* and *trans* instruments to evaluate whether the MR estimates based on the co-localizing signals corresponded amongst multiple independent methylation quantitative trait loci (mQTLs). Although many co-localizing putative signals were found including for BMD traits, the agreement between the independent mQTLs was very low (50). These results imply that many of the co-localizing signals were due to horizontal pleiotropy. Alternatively, other regions or proteins that are currently not captured by the technology may still have a causal effect.

There is growing evidence that the same gene expression level might have many different *cis* and *trans* expression quantitative trait loci (eQTLs) in different cell types and contexts. However, only a subset of those are active in the disease-relevant cell type or context and contribute to disease etiology (54). The resources used to curate eQTLs, pQTLs and mQTLs generally comprise bulk tissue and exclude bone tissue and cell types. Large scale QTL datasets derived from blood may still be useful, as osteoclasts and macrophages/monocytes originate from a common precursor. That said, the only EWAS study of BMD performed to date, based on whole blood samples, revealed negative findings (55). Novel methods to infer cell type specific DNA methylation or gene expression from bulk tissue are currently being developed (56), which may help to identify molecular signatures related to osteoporosis phenotypes. To date, osteoblast, and bone tissue eQTL datasets have only been generated in small sample sizes. However, these are currently being expanded upon, and the IFRMS knowledge portal described above is due to be updated with osteoblast-specific “omics” data in the near future. Alternatively, to gain an understanding of whether other cell types or tissues underlie GWAS signals, functional enrichment analyses across cell-type specific elements on the GWAS summary statistics can be performed (57).

## Functional Genomics: *In Vitro* Studies

Modeling the functional impact of osteoporosis *in vitro* offers a complementary approach to GWAS and *in vivo* studies, validating targets and revealing new modes of action on a cellular and molecular level. Typically, osteoblast and osteoclast cultures are employed (or precursor cell populations, e.g., mesenchymal stem cells, monocytes, respectively) to study the effect of a specific gene on cell formation and function in osteoporosis. This is achieved for example, by examining the cellular effect of gene deletion through CRISPR–Cas9 editing of candidates identified through human GWAS or *in silico* studies (58–60). Similarly, the direct use of bone cell screening assays can be used, recording evidence related to growth phenotypes, live-dead readouts, or bone cell activity and differentiation which are commonly dysregulated in human osteoporosis. This includes alkaline phosphatase levels, mineralization rates, tartrate-resistant acid phosphatase (TRAP) production, dentine resorption or alterations in key molecular markers such as Runx2, BMP2, OCN, RANKL, OPG gene expression, quantified through the use of fluorescent reporter assays, qPCR or gene array. Combination approaches assessing multiple readouts simultaneously are being explored to deliver high-throughput assessments of thousands of potential gene variants in a single experiment (61).

A major difficulty in modeling human skeletal responses and disease pathogenesis *in vitro* centres upon the cellular heterogeneity of this micro-environment. While bone-forming osteoblasts and bone-resorbing osteoclasts are most often targeted in such efforts, the complex multi-cellular bone niche consists of many more cell types including adipocytes, osteocytes, fibroblasts, stem cells and a large immune cell component. Importantly, many cell types have been linked to the onset and progression of bone disease, including osteoporosis. More accurate model systems are needed, capable of mimicking the multicellular bone environment and where the combined contribution of specific cell types and impact of genomic alterations can be more fully explored. Cellular heterogeneity within individual cell populations is being effectively probed through single cell genomic approaches, where distinct features captured at the resolution of individual cells have allowed for a more efficient isolation and characterization of cell types within the normal and osteoporotic bone marrow niche (62, 63).

*In vitro* studies also allow examination of the impact of multiple causative genomic targets operating in networks within bone cell populations, which is only beginning to be explored. A clearer understanding of the intersecting relationships between genes, and how this may contribute to a cellular osteoporotic phenotype is necessary. This may be achieved for example, by systematically analyzing a defined cellular output (e.g., growth, alkaline phosphatase) of multiple gene-pair combinations, and where gene interactions are identified by quantifying the deviation from the expected phenotype of a single-gene alteration when combined with a second (64). This allows for clusters of related genes to be characterized which may act collectively as a genomic circuit in the prevention of aberrant bone cell biology or trigger for osteoporosis pathogenesis.

## Functional Genomics: *In Vivo* Studies

### Mice

Mice are the most widely used animal model to investigate the functional role of genes identified in human genetic studies. The International Mouse Phenotyping Consortium (IMPC) aims to generate knockout mice harbouring deletions of all protein-encoding genes in a single C57BL/6N genetic background. To date, broad phenotyping of knockout mice with deletions of over 7,000 genes has been completed using the International Mouse Phenotyping Resource of Standardised Screens (IMPReSS; www.mousephenotype.org/impress/). Nevertheless, IMPReSS lacks both in depth and functional analysis of the skeleton and the IMPC thus collaborates with the Origins of Bone and Cartilage Disease (OBCD) Programme (65, 66) and the Bonebase Consortium (67) to undertake bespoke and detailed skeletal phenotyping.

The OBCD Programme uses digital X-ray microradiography, micro-CT and biomechanical testing in a rapid-throughput skeletal phenotyping pipeline to determine 19 parameters of cortical and trabecular bone structure, mineralization and strength in knockout mice compared to reference ranges obtained from >350 wild-type C57BL/6N mice. Knockout mice with abnormal parameters of both bone structure and strength (defined as >2 standard deviations away from the wild-type reference mean) are defined as having an outlier phenotype. Preliminary analysis of 1,000 knockout mice using this pipeline indicates approximately 10% display outlier phenotypes, a percentage that is broadly consistent with the >500 independent loci associated with eBMD in the recent UK Biobank GWAS (22). About 50% of mice with outlier phenotypes have deletions of genes that have not been functionally annotated to the skeleton and are not known to be related to human skeletal disease. Integration of large scale mouse phenotype data with GWAS (20, 22) and other cross-species multi-”omic” datasets (47) thus provide a rich resource to identify new genes and mechanisms involved in the pathogenesis of osteoporosis and monogenic human skeletal disorders (65, 68, 69).

### Zebrafish

More recently, zebrafish have been developed as an animal model for functional evaluation of genes linked to the skeleton (70, 71). As well as showing changes in bone density and microarchitecture (72), gene deletion can lead to bone fragility as recognized by the accumulation of fractures in the fin (73–75), and the ribs (76). Zebrafish have several advantages over mice, making experiments quicker and less expensive: they are highly fecund, laying up to 300 eggs a week; phenotypes may be evident at the larval stage (skeletal elements develop by four days); embryos develop externally, enabling genetic manipulation at the single cell stage. In addition, larvae are translucent, allowing dynamic visualization of skeletal cell behavior, for which several transgenic reporter lines are available (77, 78). Embryonic lethality is rare with fish able to survive despite mutations leading to a complete absence of bone tissue, as they are supported by water as they swim, which limits loading of malformed skeletal elements (79). As well as generating knockouts, the CRISPR/Cas9 system has proven highly efficient in zebrafish, such that a homozygous null phenotype is already detected in G0s (mosaics, crispants) in larval and adult skeleton, therefore allowing rapid screening of candidate genes (80, 81). In addition, fish scales represent a good model for performing subsequent organ cultures for drug screening (82).

## Obstacles and Opportunities

### GWAS Data Sets

Several historical obstacles to functional evaluation of genetic signals related to osteoporosis have now been overcome. For example, we now have well-powered GWAS, through which hundreds of genetic loci have been robustly identified. That said, only relatively small GWAS datasets are available relating to endophenotypes obtained using methods such as HR-pQCT, which are helpful in determining the mechanisms by which genetic pathways influence overall bone strength as reflected by BMD/eBMD. In addition, genetic studies in osteoporosis have largely been confined to cross sectional analyses, with only limited studies examining associations with longitudinal changes, exemplified by a previous look up of adult GWAS hits in BMD acquisition in adolescents (83), and a recent GWAS of pediatric bone accrual (84).

### Resources to Support Functional Studies

Osteoblast eQTL datasets based on larger samples are being generated, which will improve the accuracy of, for example, co-localization studies. The IFMRS knowledge portal is bringing together all relevant functional data, making it easier to perform functional annotation of large gene sets. Functional annotation has been advanced by characterization of the transcriptome of different cell types including osteocytes, the generation of over 7,000 knockout mouse lines, and the development of zebrafish as a rapid-throughput screening tool. However, although homology across human/mouse/zebrafish is good, there are still gaps, and difficulty in accessing bone samples to characterise expression in human tissue remains a challenge. In addition, it is technically challenging to obtain single cells from mineralized tissues, hindering evaluation of bone cell transcriptomics *in vivo*. A further limitation is that datasets available for *in silico* analysis have an inherent bias because it is only possible to analyze and interrogate genes that have already been annotated or gene functions/pathways that are already known. Furthermore, it is difficult to “quality control” the data that is interrogated. For example, there are several papers that assign different activities to PLS3 but no clear function for the protein has yet emerged and it is still unclear whether its major role is in osteoblasts or osteoclasts or both (85).

### Funding

The funding underpinning many of the resources used to support functional analyses of genetics data is finite, such as the IMPC consortium and IFMRS knowledge portal (44). In addition, given the myriad of tools available, and the range of scientific disciplines involved, the different research groups working in this area tend to pursue varying approaches. Functional follow-up of genetic signals is often performed in the context of specific projects, with the result that analyses are time- and resource limited. Funding models are generally in the form of fellowships, PhD studentships or project grants focussed on initial data collection; in contrast, it can be relatively difficult to obtain funding to support functional follow-up studies of previously collected GWAS data. Nevertheless, given the current pause in new human data collections due to the COVID-19 pandemic, arguably, greater priority should now be given to analyzing outputs of previous data collections.

## Future Directions

Given the success of genetic discovery in delivering new therapies, including anabolic treatments for osteoporosis, there is a strong case for harnessing this expanding repertoire of tools to functionally annotate the array of genetic signals for osteoporosis-related phenotypes that have already been identified. However, new strategies need to be developed to fully integrate multi-”omic” datasets with those relating to human monogenic and complex diseases, and equivalent datasets from zebrafish and mice, and potentially other species. Furthermore, it will be essential to establish large collaborative groups of experts with the necessary skillsets to harness these. Ultimately, a roadmap of functional assessments needs to be established as a coordinated effort, if the emerging wealth of genetic discoveries is to be successfully translated into new therapies for osteoporosis. The Genomics of Musculoskeletal Traits Translational network (GEMSTONE www.cost-gemstone.eu/) is a leading example of how investigators in the field from a range of different disciplines can come together to coordinate functional evaluation across genes and pathways, promote interactions between experts from different fields, and limit duplication of efforts across teams. Whereas the initial focus of GEMSTONE has been to educate and disseminate through publications and meetings, a similar approach is needed to construct an effective, multi-disciplinary, research collaboration, in order to fully exploit the exciting opportunities for pursuing functional genomics studies in osteoporosis.

## Author Contributions

All authors contributed to the paper by participating in the workshop, providing written material and/or suggestions which were incorporated into the paper, and critically appraising the scientific content. All authors contributed to the article and approved the submitted version.

## Conflict of Interest

JT has received speaker fees from UCB.

The remaining authors declare that the research was conducted in the absence of any commercial or financial relationships that could be construed as a potential conflict of interest.

## Glossary

**Table:**

EWAS	epigenome-wide association study
eQTL	expression quantitative trait locus; a genetic locus associated with gene expression (transcript) levels in a particular tissue
GWAS	genome wide association study
HR-pQCT	High resolution peripheral quantitative computed tomography
MicroCT	Micro computed tomography
mQTL	methylation quantitative trait locus; a genetic locus associated with DNA methylation levels in a particular tissue
pQCT	peripheral quantitative computed tomography
pQTL	protein quantitative trait locus; a genetic locus associated with protein levels in a particular tissue
